# Fine-Tuning Enhancer Models to Predict Transcriptional Targets across Multiple Genomes

**DOI:** 10.1371/journal.pone.0001115

**Published:** 2007-11-07

**Authors:** Stein Aerts, Jacques van Helden, Olivier Sand, Bassem A. Hassan

**Affiliations:** 1 Laboratory of Neurogenetics, Department of Molecular and Developmental Genetics, Vlaams Instituut voor Biotechnologie (VIB), Leuven, Belgium; 2 Department of Human Genetics, K.U. Leuven School of Medicine, Leuven, Belgium; 3 Service de Conformation des Macromolécules Biologiques et de Bioinformatique, Département de Biologie Moléculaire, Université Libre de Bruxelles, Bruxelles, Belgium; Genome Institute of Singapore, Singapore

## Abstract

Networks of regulatory relations between transcription factors (TF) and their target genes (TG)- implemented through TF binding sites (TFBS)- are key features of biology. An idealized approach to solving such networks consists of starting from a consensus TFBS or a position weight matrix (PWM) to generate a high accuracy list of candidate TGs for biological validation. Developing and evaluating such approaches remains a formidable challenge in regulatory bioinformatics. We perform a benchmark study on 34 *Drosophila* TFs to assess existing TFBS and *cis*-regulatory module (CRM) detection methods, with a strong focus on the use of multiple genomes. Particularly, for CRM-modelling we investigate the addition of orthologous sites to a known PWM to construct *phyloPWMs* and we assess the added value of phylogenentic footprinting to predict contextual motifs around known TFBSs. For CRM-prediction, we compare motif conservation with network-level conservation approaches across multiple genomes. Choosing the optimal training and scoring strategies strongly enhances the performance of TG prediction for more than half of the tested TFs. Finally, we analyse a 35^th^ TF, namely Eyeless, and find a significant overlap between predicted TGs and candidate TGs identified by microarray expression studies. In summary we identify several ways to optimize TF-specific TG predictions, some of which can be applied to all TFs, and others that can be applied only to particular TFs. The ability to model known TF-TG relations, together with the use of multiple genomes, results in a significant step forward in solving the architecture of gene regulatory networks.

## Introduction

The characterization and understanding of gene regulatory interaction networks that rigorously control the execution of genetic programs that make functional cells, tissues, and organisms is a key challenge for post-genome biology. Such regulatory interactions are formed by transcription factors (TFs) and their target genes (TGs) and are implemented via TF DNA-binding sites (TFBS) located in *cis*-regulatory modules (CRM) of TGs. A CRM is a promoter or enhancer sequence that contains TFBSs for one or more TFs and that controls a specific aspect of the expression pattern of the TG [1]. A consequence of genetic pleiotropy- one gene, multiple functions- is that genes often have several distinct expression patterns regulated by several distinct CRMs per gene. For example, the expression of the *atonal* TF in *Drosophila melanogaster* (*Dmel*) in different tissues is regulated by discrete CRMs, some of which are also autoregulatory [2]–[4]. It is therefore not surprising that comparative genomics and computational CRM predictions [5] suggest large numbers of CRMs per genome, implying very large numbers of regulatory interactions. The vast majority of these interactions remain to be discovered. This complexity means that it will be practically impossible to understand the logic and organization of gene regulatory networks without the application of genome-wide, TF-specific computational TG discovery methods. Although genetic interaction, expression profiling and chromatin binding approaches can provide lists of candidate TGs, they each suffer from disadvantages such as high cost, technical limitations, inability to detect direct TGs, and prohibitive numbers of conditions to test [6]. Therefore, experimental approaches would benefit greatly from being complemented by *in silico* TG discovery methods.

Ideally, computational approaches to mine entire genomes for TFBSs and CRMs would generate highly accurate lists of candidate TGs that can be taken directly to *in vivo* biological validation. Bioinformatics approaches have been developed to predict TFBSs for TFs that have a known consensus TFBS or position weight matrix (PWM). Unfortunately, this approach results in a 1000-fold excess of false predictions when applied on a genomic scale [7]. In a handful of cases, however, genome-scale scanning for TF-target interactions has been successful, particularly if the binding sites are evolutionarily conserved and if they occur in clusters. These can be either homotypic clusters with multiple TFBSs for one TF as is the case for *Drosophila* Dorsal [8], Bicoid [9] and Suppressor of Hairless [10], or heterotypic TFBS clusters for several very well characterized TFs [11]–[13]. The methods applied in these studies are often based on Hidden Markov Models (HMM) [14]–[16]. They take one motif or a set of motifs - in the form of PWMs - as input and identify confined regions in large DNA sequences that harbor significantly more motif instances than expected by chance or than given by a background model. Alternatively, predicted TFBSs can be filtered by their occurrence in conserved non-coding DNA stretches [17].

There are a few reasons why such methods cannot be generalized. First, PWMs for most TFs are built from few instances and the minimal number of instances for a useful PWM is not known. Second, not all TFs regulate their TGs via homotypic TFBS clusters. Third, for most CRMs that contain heterotypic clusters the cooperating TFs, let alone their PWMs, are unknown. Fourth, several independent studies have found that sequence conservation *per se* is not sufficient to identify enhancers because many enhancers are functionally conserved without sharing high levels of sequence identity across a long DNA stretch [11], [13], [18]. Based on this latter point, methods have been developed to search for conserved motif clusters across two genomes [13], [19]. However, these methods have not been assessed for their ability to score more than two genomes, nor for their performance on a wide range of TFs. Taken together, these limitations mean that it is currently unclear under what conditions, using which parameters and for which TFs genome-scale TG prediction is feasible.

To investigate these issues we perform a benchmark study on genome-scale TG prediction for individual TFs. The benchmark consists of *in silico* validations on known TGs with identified TFBSs for 34 Drosophila TFs from the FlyReg database [20]. The availability of the full genome sequences of twelve *Drosophila* species at a range of evolutionary distances from *D. melanogaster* provides the opportunity to study the evolution of genes [21] and the discovery and annotation of functional elements like protein-coding genes, miRNA genes, and regulatory motifs [22]. In our benchmark study, we take advantage of the multiple genomes in several ways. First, we compare the use of *Dmel* PWMs built from the known binding sites versus *phyloPWMs* built from orthologous binding sites from 10 other Drosophila species. Next, we exploit all *Drosophila* genomes to improve the prediction of homotypic TFBS clusters, either by applying motif conservation or network-level conservation filters. For TFs that show a low performance in this approach, we investigate the use of heterotypic enhancer models consisting of de novo discovered motifs by phylogenetic footprinting, also using all *Drosophila* genomes. Finally, we model the known TGs of a 35^th^ TF not included in the initial assessment, namely the eye determination TF Eyeless (Ey). We find a significant overlap between predicted TGs and a list of candidate targets obtained from a recently published and biologically validated microarray experiment.

An important conclusion of this study is that no general rule exists that applies to all, or even most, TFs. However, most TFs benefit greatly from the use of multiple genomes in enhancer scoring. Also, we find that by performing cross-validations, the optimal strategy and parameters can be determined for each TF. By training these parameters and through the extensive use of multiple genomes, combined with Gene Ontology filters or microarray data, we estimate that genome-wide discovery of TGs is feasible for about 50% of the TFs tested.

## Results

### Detecting homotypic enhancers using known PWMs while eliminating validation bias

**Figure 1:**
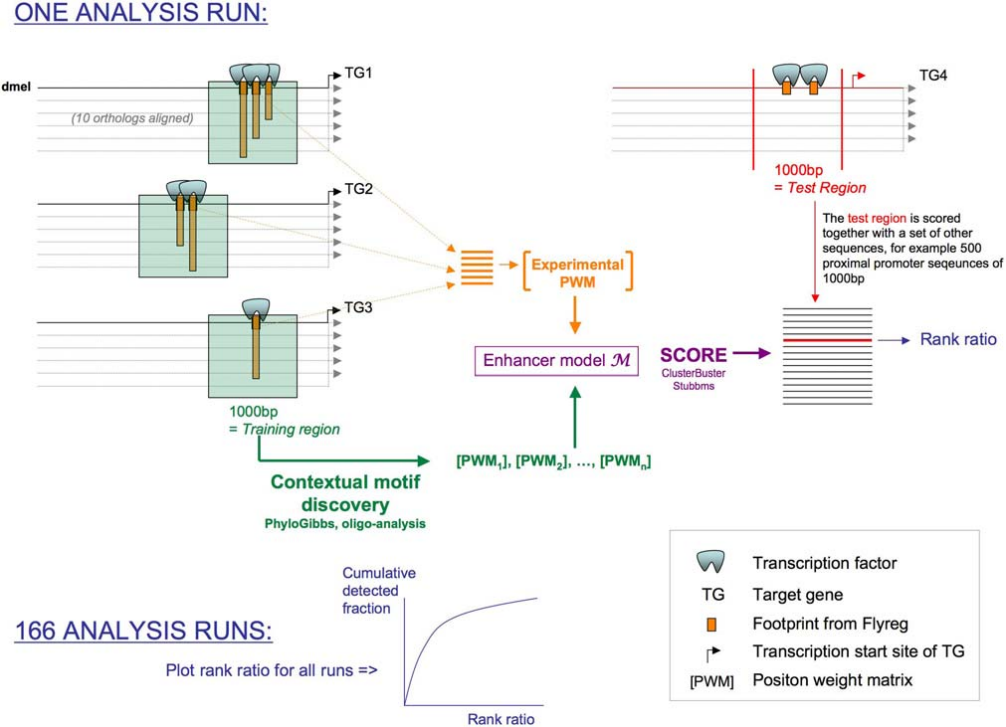
LOOCV assessment scheme. An enhancer model, consisting of one or more PWMs, is trained on known target regions of a TF, excluding the target region of the test TG (here TG4 is excluded). This left-out region, together with a set of negative sequences, is scored with the enhancer model. All 166 rank ratio's are plotted cumulatively.

**Figure 2:**
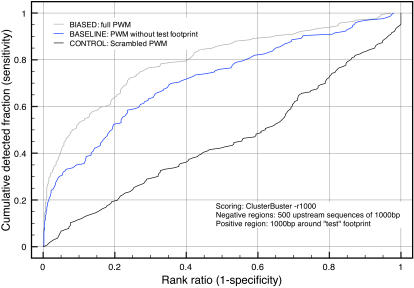
LOOCV performance for homotypic cluster detection on *Dmel.* For each of the 34 TFs, target sequences are scored with Cluster-Buster, using a PWM built from all known binding sites (grey curve) or from all known binding sites except those located in the region being scored (blue curve). Scrambled PWMs are used as negative controls (black curve).

The first, and most straightforward, strategy for motif-based TG prediction, is to use an existing consensus site or PWM for the TF under study. The genome-wide discovery of TGs through homotypic cluster prediction [23] was already shown to be feasible for a number of TFs, particularly Dorsal [8], Bicoid [9] and Suppressor of Hairless [10]. Here, we test this strategy for all 34 TFs in our dataset (see Methods and Table S1). We have chosen the Hidden Markov Model implementation of Cluster-Buster [15], although other methods that take a PWM as input are available [16], [24], [25]. Through leave-one-out cross-validation (LOOCV), we test whether a 1000 bp ‘positive’ sequence flanking one or more known binding sites can be discriminated from ‘negative sequences’ by the motif cluster score. As negative sequences we use 500 randomly selected proximal promoter sequences. In each run, the 501 sequences are ordered by descending score and the rank of the positive region is recorded (Figure 1). For one TF, this process is repeated *f* times, each time using a different target sequence as the positive region. The process is also repeated *t* times, once for each TF. This way, 166 rank positions (Σ*f_i_t_i_*) are obtained. Rank positions are then plotted cumulatively to yield a special type of Receiver Operating Characteristic (ROC) curves. For different analysis methods or parameter settings, different curves are obtained that can be compared, as done previously for related and different problems [26]–[30]. The area under this curve (AUC) is a measure of the overall detection performance integrating both sensitivity and specificity values. Using the approach outlined above, we first asked if searching for homotypic clusters of TFBSs is a generally applicable approach for detecting TGs. A PWM is built from all the TFBSs of a specific TF in the dataset, including the TFBSs present in the left-out region. As a negative control, we use scrambled PWMs. Compared to the negative control (Figure 2, black curve), the approach yields relatively strong average performance across the 34 TFs (Figure 2, grey curve). In other words, it is possible, at least to some extent, to distinguish the test sequence from other sequences when the footprints that are present in the test sequence are used to build the training PWM. Although this is the standard approach used in almost all studies, there is a serious problem with it: it introduces a strong bias because the TFBSs in the test region are contained within the training PWM. As such, this approach does not reflect the ‘real life’ situation in which biologists are attempting to discover novel TGs starting from known ones. To simulate a realistic situation, we excluded all TFBSs that are contained within the test sequence from the training PWM. The unbiased performance curve that is obtained when leaving-out the sites in the test regions is our baseline (Figure 2, blue curve). Whether low-complexity regions in test and negative sequences were masked or not did not influence this overall curve (data not shown), and different sets of negative sequences, for example known enhancers from REDfly [31] or genomic flanking regions, also gave similar results (Figure S1). Not surprisingly, the AUC for this curve is smaller than for the biased curve, but it remains significantly larger than the negative control.

**Figure 3:**
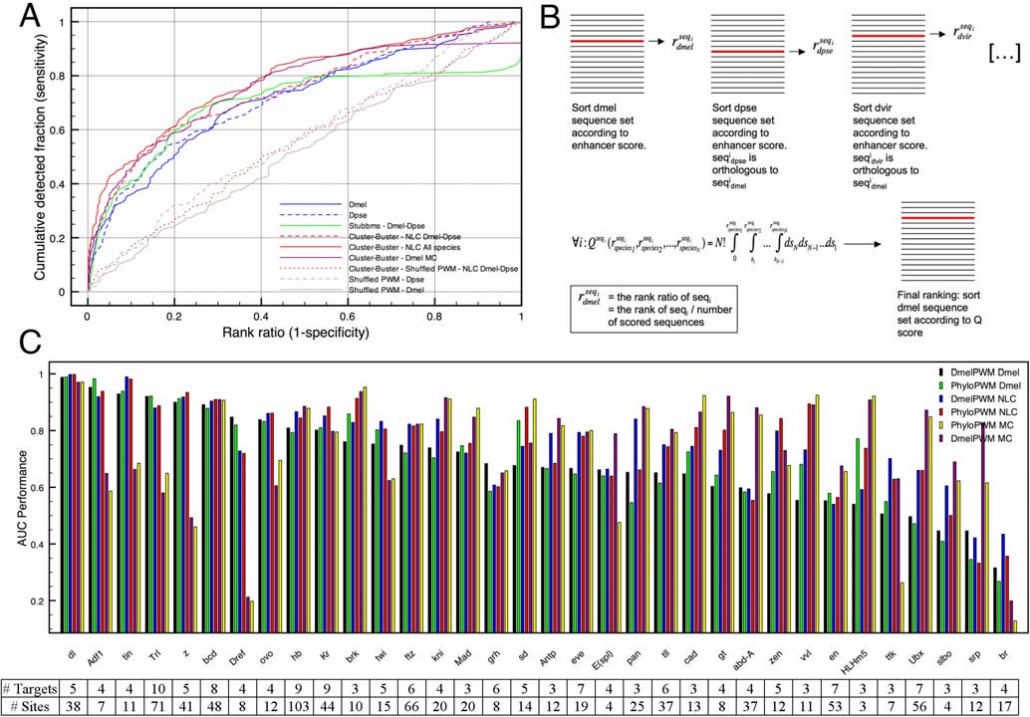
Integrating HMM-based enhancer scoring across multiple genomes. (A) LOOCV performances of Cluster-Buster (red dashed curve) and STUBBMS (green curve) on two genomes (*Dmel* and *Dpse*), Cluster-Buster on all genomes using network-level conservation (NLC) (red curve), and Cluster-Buster combined with motif conservation (MC) (purple curve). The red dotted curve is the negative control. (B) Implementation of network-level conservation by integrating the Cluster-Buster scores on multiple genomes through order statistics. Rank ratios for orthologous sequences (both positive and negative) are obtained for each species separately and are integrated by the order statistics formula. *Dmel* sequences are finally ranked according to the integrated score. (C) LOOCV performances (AUC values in the y axis) for each TF (x axis), using the *Dmel* PWM or the phyloPWM. The first two bars represent the scoring on *Dmel* alone, the next two on all genomes with network-level conservation (NLC), and the last two on all genomes with motif conservation (MC). The TFs are sorted according to decreasing baseline performance (black).

Next we examined the performance of unbiased homotypic cluster detection in detail. We find that 37 out of 166 test regions rank in the top 2% (10/501 scored sequences) representing 19 of 34 TFs. To investigate the performances of each individual TF in more detail, we plot ROC curves per TF and calculate AUC scores for each TF separately (Figure 3C, black bars). Amongst the high scoring TFs are Dorsal (AUC = 0.99) and Bicoid (AUC = 0.89) that were previously known to use homotypic clusters. In addition, the homotypic cluster approach to TG prediction works very well for Adh transcription factor 1 (0.95), Tinman (0.93), Trithorax-like (0.92), and Zeste (0.90). We could not find any obvious correlation between the performance and the size of the data set (Figure 3C). Amongst the high-performing TFs are both factors with large data sets (e.g., dl, Trl, bcd) and factors with small data sets (e.g., Adf1, tin, Dref). Similarly, amongst the low-performing TFs are factors with large (e.g., en, Ubx) and small (e.g., HLHm5, slbo, srp) datasets.

### Adding orthologous sites can improve the quality of sparse PWMs

**Figure 4:**
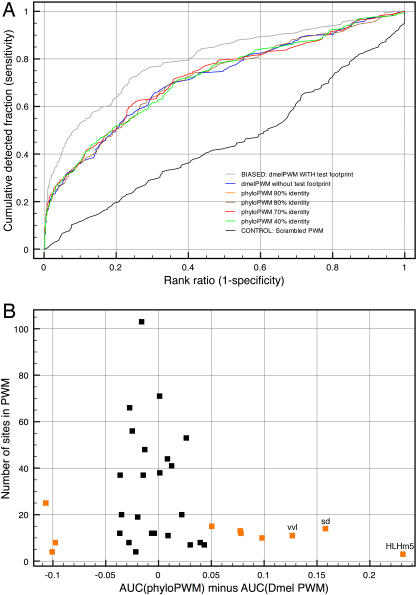
Homotypic cluster detection with *phyloPWMs.* (A) LOOCV performance for *Dmel* PWMs (blue curve), and phyloPWMs from all species tested (*Dmel, Dsim, Dsec, Dere, Dyak, Dana, Dpse, Dper, Dvir, Dmoj,* and *Dgri*). Orthologous sites were chosen based on a minimum of either 40% (green curve), 70% (red), 80% (brown), or 90% (orange) identity with the *Dmel* true binding site. (B) Differences between the AUC values obtained from a *Dmel* PWM and a phyloPWM, for each TF. TFs with differences above 0.05 are colored orange. PWMs with few sites (y-axis) have greater AUC differences than PWMs with many sites.

Given the availability of multiple *Drosophila* genomes, an interesting question is whether better PWMs can be built using sequences that are orthologous to the known TFBSs. The issue of phylogenetic conservation of TFBSs is paradoxical because on the one hand, TFBSs are known to have a high evolutionary turn-over rate [32], while on the other hand many have been discovered by virtue of their evolutionary conservation. We asked if the addition of conserved sites- defined as aligned sites sharing more than 40%, 70%, 80%, or 90% identity between a given *Drosophila* species and *Dmel*- to the PWM improves the overall performance (see Methods). *A priori* we expected the 70% identity cut-off to perform best because we calculated from all PWMs in the TRANSFAC library that the sites that make up a PWM have 72% identity on average. This cut-off allows one substitution in a 6 bp motif, two in a 8 bp motif, and three in a 10 bp motif. We expected that a less stringent cut-off (e.g., 40%) would allow too many negative sequences in the phyloPWM, and that a more stringent cut-off (e.g., 90%) would not introduce enough variability. To our surprise, we do not observe an increased average performance across all 34 TFs with any of these phyloPWMs (Figure 4A). This is not because we have too few conserved sites due to alignment errors [32] since the 70% phyloPWMs for example are built from 7.81 times more sites on average than the *Dmel* PWM (where the maximal number of orthologous sites would be 11 times the number of Dmel sites, for 11 genomes used). When looking at individual TFs, we find that only three TFs benefit from a phyloPWM with more than 10% performance increase, namely HLHm5, Scalloped, and Ventral Veins Lacking. Figure 4B shows the difference in AUC for each TF between the *Dmel*-PWM and the phyloPWM. PWMs built from fewer than 20 sites are found to be more susceptible to phylogenetic extension than PWMs built from more than 20 sites (Figure 4B). Note that the performance of a PWM with few sites can also decrease when adding orthologous sites. This is a first illustration of how the cross-validation assessment can assist in the selection of the most appropriate model, in this case the type of PWM, for each TF.

### Different approaches for enhancer scoring using multiple genomes: network-level conservation versus motif conservation

Using multiple genomes in the training step to construct PWMs only sometimes improve detection of test TG enhancers. However, using sequence conservation between *Dmel* and *D. pseudoobscura (Dpse)* during enhancer scoring has been suggested as a useful filter [13], [19]. The program STUBBMS [19] also implements a Hidden Markov Model like Cluster-Buster used above, but can score two genomes simultaneously given one or more PWMs as input. When we apply the program STUBBMS [19] to the combined scoring of *Dmel* and *Dpse* regions we find a better performance than using Cluster-Buster on *Dmel* alone (Figure 3A). This suggests that the addition of a second genome improves enhancer scoring. However, STUBBMS is limited to only two genomes. To solve this we propose two new approaches. In the first approach we attempt to integrate HMM-based enhancer scoring with ranking across muliple genomes using the premise that orthologous enhancers can be functionally conserved without necessarily having high sequence identity [11]. To this end we scored each species separately with Cluster-Buster and used order statistics [30], [33] to combine the species-specific rankings of all orthologous test regions into one overall rank (Figure 3B and Methods). This approach can be classified under the so-called network-level conservation approaches [34]. In our case, sequence alignment data are only used initially to search for the best orthologous match(es) in a given species to a *Dmel* 1 kb region. We assembled orthologous sequences from 10 other species by directly querying the net alignments of the UCSC Genome Browser database [35]. We also constructed a second series of sets using LAGAN [36] aligments and these yielded similar performance results (data not shown).

We find that using order statistics to integrate information from all 11 species during enhancer scoring for homotypic clusters results in the best performance, with a significant improvement over *Dmel* alone, or any pairwise combination with *Dmel* (data shown for *Dmel*-*Dpse*). To ensure that the order statistics themselves do not bias the results, we combined the rankings obtained from scrambled PWMs. This did not result in an increased performance compared to scrambled PWMs from *Dmel* alone (Figure 3A, red dotted curve). Therefore, including all *Drosophila* genomes in the scoring step improves enhancer detection when searching for homotypic clusters.

In a second, more classical approach, we filtered the HMM-based motif cluster predictions based on sequence conservation of the predicted motifs themselves. Using the phastCons scores [37] from the UCSC browser we masked all nucleotides with a conservation score below 0.90 before applying the HMM-scoring (thresholds of 0.5 and 0.7 gave similar results; data not shown). The resulting overall performance is better than the STUBB-MS performance on two genomes only (purple curve in Figure 3B), but slightly worse than the performance of the network-level conservation approach.

Importantly, the ROC curves depict averages across 34 transcription factors. When looking at the performances of the individual TFs, it became clear that some factors benefit more from network-level conservation (e.g., tin, kr, twi, zen) and others are better suited for motif conservation applications (e.g., abd-A, Ubx, HLHm5, cad, gt). Again, we could not find any obvious correlation between the methods and the size of the starting data sets, neither in terms of target genes nor in terms of binding sites per TF. The TF-wise performances for each method, both for the Dmel PWM and the phyloPWM are presented in Figure 3C, together with the size of each data set. This is a second illustration of how the cross-validation assessment can assist in the selection of the most appropriate model, in this case the type of conservation filter.

### Learning contextual motifs to construct heterotypic enhancer models

Using multiple genomes resulted in improved performance in homotypic cluster prediction for many TFs. However, it is known that many TFs operate in cooperation with other TFs that bind to different binding sites in the neighborhood, which is often a relatively small sequence window (e.g., 1 kb). To take the existence of such heterotypic motif clusters into account in our dataset of 166 enhancers, we added a pattern discovery step to identify a shared context between all regions targeted by the same TF, always excluding the left-out test region (Figure 1). The trained model consists of a set of motifs, represented by PWMs, used to score the left-out region. As for homotypic enhancer models, a large set of negative sequences are scored and the rank of the test region within this test set is plotted.

**Figure 5:**
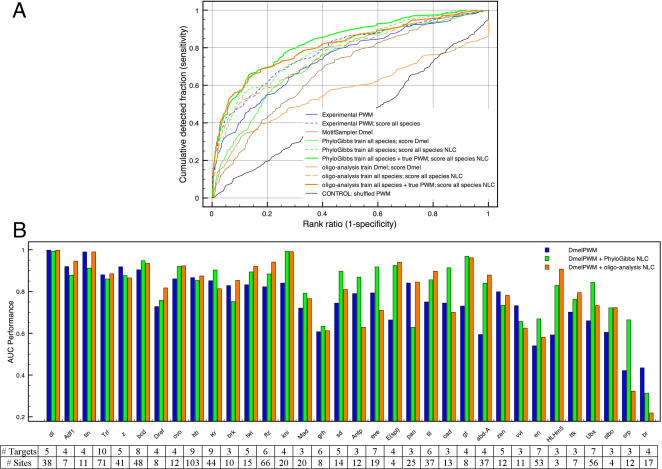
LOOCV performances for heterotypic CRM-models. (A) Heterotypic models consist of PWMs obtained by motif discovery on *Dmel* sequences using MotifSampler (brown curve) or oligo-analysis (orange curve) and on all species using PhyloGibbs (green curve). Models consisting of combined *de novo* PWMs with the true experimental PWM are shown for oligo-analysis (dashed orange) and PhyloGibbs (dashed green). Scoring is either done on *Dmel* alone (thin full lines) or on all species by network-level conservation (NLC) (thick lines and dashed lines). (B) In green and orange are LOOCV performances for scoring with heterotypic models (either species-specific for oligo-analysis or cross-species for PhyloGibbs) on all species including the *Dmel* PWM (cfr thick full lines in A). Scoring on all species is done by network-level conservation (NLC). In blue is the control, namely NLC using the *Dmel* PWM alone, without newly discovered motifs.

First, we apply two traditional motif discovery methods, namely MotifSampler [38] and oligo-analysis [39] to each *Dmel* training set. The number of motifs found by MotifSampler is a parameter of the method, and was set to 5. Oligo-analysis has the advantage that only significantly over-represented k-mers (where k is 6,7 or 8) are reported, yielding between zero and 18 motifs per regulon, with an average of 4.3 and a standard deviation of 4.17. Oligo-analysis gives slightly better results than MotifSampler in the high-specificity range in which we are interested. The reason why the performance for oligo-analysis decreases in the low-specificity range is our conservative approach that instructs Cluster-Buster to rank the left-out region last when oligo-analysis does not find any common motifs in the training set. Note that oligo-analysis yields over-represented DNA words, not PWMs, while Cluster-Buster requires PWMs as input for the scoring step. To solve this problem we transform each over-represented word into a pseudo-PWM (see Methods and Supplementary Note 1). In agreement with published results [40], we observe that single species motif discovery results in unsatisfactory enhancer models that are not able to generalize (Figure 5A). We therefore reasoned that a multiple species motif discovery method might increase performance. We chose PhyloGibbs [41] but other methods like PhyME, OrthoMEME, PhyloConn are also available. Similar to MotifSampler, the number of motifs found by PhyloGibbs is a parameter of the method and was set to 5. The performance of the resulting enhancer models (green curve in Figure 5A) is slightly better than those trained by oligo-analysis on *Dmel* alone, but remains unsatisfactory. When inspecting the predicted motifs by the pattern discovery methods, we observed that even the real motif can only be found in a limited number of cases, for example for 8 out of 34 factors when using PhyloGibbs (Figure S2), and for 14 out of 34 factors when using oligo-analysis (data not shown). To further improve the performance, we combined the known, experimentally determined, PWM with the *de novo* discovered PWMs in one enhancer model. The average performance of this hybrid model across all TFs is similar to that of the homotypic baseline model (Figure 5A, dashed green and orange curve for PhyloGibbs and oligo-analysis respectively). In an attempt to further increase the sensitivity and specificity, we combined the search for heterotypic clusters with our new method of scoring multiple genomes using network-level conservation. To do this we applied motif discovery with either PhyloGibbs or oligo-analysis. For PhyloGibbs we use the same PWMs for all species because each PWM was built from all species during the PhyloGibbs motif discovery step. For oligo-analysis, which works on single species, we discovered new motifs in each species separately and then scored each species with its own species-specific collection of pseudo-PWMs. The performance curves for the heterotypic models consisting of PhyloGibbs or oligo-analysis motifs, combined with the true PWM, and scored on all available species show significantly improved performance (Figure 5B). Of the 166 enhancers 49 (29.5%) and 54 (32.5%) rank within the top 2% for models from PhyloGibbs and oligo-analysis, respectively.

We examined individual TF performances (Figure 5B) and compared them to the performances of the *Dmel* PWM with network conservation (i.e., the red bars in Figure 3C) because here the experimental PWM and the scoring method are the same. We find several factors, such as gt, kni, E(spl), tll, ftz, abd-a, and HLHm5 with significantly improved performances. This is the third illustration of how cross-validations can be used to identify the optimal enhancer model for each transcription factor.

When the optimal method is used for each TF, the AUC increases by more than 10% over the initial performance baseline for 25 out of the 34 TFs. The number of TFs for TG detection is highly performant (AUC>0.9) increases from 5 to 19 out of 34 (Figure 3C, black bars and Figure 5B).

### Genome-wide target gene prediction

The ultimate goal of computational target gene prediction is to obtain a high quality set of candidate targets by scanning one or more genomes. The cross-validations described above show promising results for a number of transcription factors. However, in practice several obstacles arise. The first obstacle in genome scanning is the definition of the search space and the association of a predicted regulatory region with a target gene. For the *Drosophila* genomes we chose to attribute a CRM to a gene when it either lies in an intron of that gene or within 5 kb upstream of that gene. Each sequence region of a gene (i.e., each intron and the upstream sequence) receives the maximal CRM score found within that region. For the network-level conservation approach, each region in the *Dmel* genome is associated with its orthologous region in another genome using UCSC's genome alignments. The top 100 scoring genes for each of the 34 TFs (using the optimal enhancer model for each TF), together with the locations of the predicted motif clusters, are presented on our website (http://med.kuleuven.be/cme-mg/lng/cisTarget).

The second, more problematic obstacle is the size of the test set that now consists of 93330 regions (all regions for all genes). Sensitivities of 50% will no longer be feasible because the top 10% regions represent more than 1000 genes, which is too large for biological validation and even for further filtering by functional annotations. On the other hand, we know from the LOOCV tests that even the top 1% (136 genes) could contain a few *bona fide* target genes, because about 10% of the targets (18 out of 166 for the phyloPWM with motif conservation) were found in the top 1% (i.e., top 5 out of 501 regions) in the LOOCV. We attempted to enrich for true targets within the top 100 genes by comparing over-represented Gene Ontology (GO) terms with the GO annotation of the TF itself. When the optimal model – based on the LOOCV results - is chosen for each TF, 15 TFs show a top 100 TG prediction with a significant enrichment of at least one biological process of the TF (see Table 1; the complete GO results for all TFs are available from our website). To test whether the selection of the optimal model is important for GO enrichment, we compared the optimal model to one reference model, namely the homotypic model using the *Dmel* PWM and network-level conservation. Indeed, by using the reference model instead of the optimal model for each TF, 8 factors loose the GO enrichment that was relevant for the TF, another three TFs have a p-value that is still significant but less than the optimal method, one TF has the same p-value and only 1 TF has a better p-value (data not shown).

**Table 1:** Selection of GO-filtered genome-wide target gene predictions from our website.

TF	Known TGs	Model	AUC	GO ID	GO Term	P-value	Candidate TGs
bcd	tll, eve, ems, Kr, kni, salm, h, hb	Homotypic P-NLC	0.91	GO:0008595	determination of anterior/posterior axis, embryo	2.00E-07	gt, kni, pum, hb, slp1, oc, tll, eve, Kr
dl	rho, zen, twi, sna, dpp	Homotypic F-NLC	1	GO:0007498	mesoderm development	6.08E-04	dpp, mbl, zfh1, sna, S, pnt, twi, tmod, jeb, vnd
tin	Mef2, eve, betaTub60D, tin	Homotypic F-NLC	0.99	GO:0007507	heart development	4.63E-11	mid, G-oalpha47A, fz, apt, lbl, svp, hh, fas, tin, Mef2, pnr
brk	zen, lab, bi	Homotypic P-MC	0.95	GO:0007179	transforming growth factor beta receptor signaling pathway	0.0308	Dad, sog, pnr, bun
cad	ftz, kni, salm	Homotypic P-MC	0.92	GO:0007379	segment specification	7.58E-06	kn, osa, Antp, cad, Abd-B, kis, abd-A
pan	slp1, eve, Ser	Homotypic P-MC	0.88	GO:0016055	Wnt receptor signaling pathway	0.0168	osa, Notum, Wnt4, Axn, par-1
Mad	zen, vg, tin	Homotypic P-MC	0.88	GO:0007267	cell-cell signaling	0.00563	DopR, para, NetA, pum, mib1, D2R, shot, wb, scrib, Or98a, bab1, dlg1, fru
sd	ct, bs, vg, kni, salm	Homotypic P-MC	0.91	GO:0007476	wing morphogenesis	0.03205	vg, dpp, bs, fz, shot, sgg, px
E(spl)	ac, sc, l(1)sc, Espl	Heterotypic F-O-NLC	0.94	GO:0045165	cell fate commitment	0.000383	fz, l(1)sc, Ubx, pum, vn, bun, spen, mam, fas, sc
gt	abd-A, eve, Kr, kni	HeterotypicF-O-NLC	0.96	GO:0007354	zygotic determination of anterior/posterior axis, embryo	0.00104	tll, gt, kni, sog, Kr
HLHm5	ac, l(1)sc, Espl	Heterotypic F-O-NLC	0.91	GO:0045165	cell fate commitment	4.40E-05	hth, srp, pnt, l(1)sc, pum, Ubx, vn, bun, spen, ac, kay
kni	Ubx, eve, Kr, h	Heterotypic F-O-NLC	0.99	GO:0035290	trunk segmentation	0.00150	kni, Ubx, eve, Kr
ovo	orb, otu, Sxl, ovo	Heterotypic F-O-NLC	0.92	GO:0009993	oogenesis (sensu Insecta)	0.00158	dpp, Ptp61F, Eip74EF, Sxl, pum, bun, dnc, ovo, Fas3, ttk, sty, Eip75B, Mef2, ct
tll	Ubx, ems, Kr, kni, h, hb	Heterotypic F-O-NLC	0.9	GO:0045165	cell fate commitment	1.76E-09	hth, eya, fz, kni, vvl, Ubx, pum, hdc, run, h, tll, fas, sc, Kr, ct
twi	rho, Ubx, sna, sim, tin	Heterotypic F-O-NLC	0.92	GO:0007507	heart development	0.00244	fz, fas, Antp, apt, Ubx, Mef2
ey	so, shf, Optix, eya, ato	Homotypic F-NLC	0.94	GO:0007456	eye development (sensu Endopterygota)	9.98E-05	hth, Optix, Fas2, eya, fz, pnt, so, bun, toy, lilli, S, klar, fred
				Overlap with 188 upregulated genes after ey over-expression from [43]		2.33e-05	mspo, SK, so, toy, ey, CG17816, Optix, CG30492, CG32521, osp, Fas2, CG5888, Tie, eya

Candidate targets are presented for those TFs with AUC above 0.88 and with a TF-associated functional enrichment in the list of top 100 candidates. Results for other factors, for other functional classes, and for genomic locations of predicted motif clusters can be found at http://med.kuleuven.be/cme-mg/lng/cisTarget/. F = FlyReg PWM, P = phyloPWM, NLC = Network-level conservation, MC = Motif Conservation, F-O = FlyReg PWM+oligo-analysis motifs.

Filtering based on functional annotation is however limited to the detection of genes with GO IDs. For genes without GO IDs computational predictions can be complemented with data from other sources, such as phenotypic data, protein-protein interaction or expression data. As an example we analyzed a regulon of the eye determination transcription factor Ey. Ey was not included in our earlier dataset because the FlyReg database only contains one target gene of Ey, namely *sine oculis* (*so*). However, recent studies have identified four more targets of Ey: *eya*, *shf*, *Optix*, and *atonal* [42]. The first three were found by expression studies comparing wild type and ectopic Ey over-expression [43]. All five genes have identified Ey binding sites that have been validated through *in vitro* binding studies and *in vivo* reporter assays. Ostrin *et al.* conducted a microarray experiment and identified 188 potential TGs of Ey whose expression is independent of the function of the retinal differentiation TF *atonal*. The total of five target regions can now be subjected to cross-validation. The assessment suggests that the homotypic approach with multiple genomes performs best for Ey, although the differences between methods are small in the high specificity range in which we are interested (data not shown). Although Ostrin *et al*. used a phyloPWM in their study [43], the cross-validation performances we observed for our phyloPWM were similar to the *Dmel* PWM. The homotypic model using the *Dmel* PWM and network-level conservation was applied to the full genome, ranking all 5 kb upstream regions and all introns. The top 100 scoring genes have “photoreceptor cell differentiation” over-represented with a p-value of 6.45e-06. The 9 photoreceptor differentiation genes in the top 100 are *bun, eya, fz, hth, lilli, mbl, Optix, pnt, and sdk*. It is important to note that neither GO:0048748, nor any other eye development GO term, is over-represented in the top100 candidate genes when the scoring is performed on *Dmel* alone.

There are 9 genes in the top 100 genes that are also present in the set of 188 genes of Ostrin *et al*. To determine the significance of this result, the same procedure was repeated for 100 randomizations of the Ey PWM. The top 100 scoring genes from randomized PWMs contain 3.70±2.84 genes (95% confidence interval) in common with the Ostrin set of 188 genes. The maximal difference between the true ranking and the randomizations is obtained at a threshold of 171 genes (Figure S3). At this threshold, the randomized PWMs yield an overlap of 6.05±3.65 while the true PWM yields an overlap of 14 genes (p-value of 2.3288e-05). These 14 genes are *mspo, SK, so, toy, ey, CG17816, Optix, CG30492, CG32521, osp, Fas2, CG5888, Tie,* and *eya*.

## Discussion

Gene regulatory networks of TFs and their TGs play key roles in development, homeostasis and behavior. The relationship between a TF and its TGs is achieved through “DNA words”, usually 4–12 bp long, acting as binding sites for the TF in question. A collection of such motifs regulating a specific aspect of the expression of a gene defines a CRM. Therefore, to understand network organization, we need to understand the organizational logic of CRMs. Since most networks mediating a specific biological function consist of multiple TFs and their- sometimes overlapping- TGs, the capacity to detect TGs genome-wide and *in silico* with high accuracy would bring major advantages. In this respect, regulatory bioinformatics faces a few challenges. First, the organizational logic(s) of CRMs is not clear. Do most TFs regulate their TGs via multiple or single TFBSs? How conserved do such TFBSs need to be at the sequence level to be detectable? Can the contextual information around a given TFBS in a CRM be harnessed to improve TG detection? Does the availability of multiple related genomes foster or complicate CRM detection? And finally, do different TFs follow different logics? Developing and assessing methods that can address these issues is clearly a major goal of regulatory bioinformatics.

To begin to address these issues we took advantage of the availability of 12 *Drosophila* genomes and the *Drosophila* database of TF-TG relations (FlyReg) to perform a benchmark study on genome-wide *in silico* TG prediction. *Drosophila* is ideally suited for such an approach not only because multiple genomes are now available, but also because a few regulatory networks, such as the segmentation network, have been well-established *in vivo* resulting in several deeply understood TF-TG relations. One challenging problem of target gene prediction is the mapping of an *in silico* predicted regulatory locus to the gene it potentially regulates. Indeed, several known enhancers are located one or several genes upstream of the actual target gene, for example the cluster of dorsal binding sites that regulates zen is located directly upstream of CG1162. To circumvent this problem in the benchmark analysis and to make direct comparisons at the sequence level possible, we added isolated regulatory regions to sets of negative sequences. This setup has the additional advantage that it requires less computational resources so that more parameters can be varied. We chose the same region size for each enhancer and each transcription factor to exclude confounding effects of the size when comparing across factors (Cluster-Buster can generate higher scores for larger regions). For the cross-validations we arbitrarily set this size to 1 kb. Such a choice is justified because the cross-validations are intended to compare relative performances rather than absolute performances. When a cross-validation procedure is applied on a single factor under study, the region size could be considered as an additional parameter. For the genome-wide scoring, the size of the motif clusters is determined by Cluster-Buster.

Cross-validation tests and subsequent genome-wide TG predictions result in both higher average performances across a data set of more than 30 TFs (of a total of approximately 700 TFs in the *Dmel* genome [44]) as well as determination of the optimal parameters for each of the individual TFs. This is of particular value for molecular geneticists who are likely to be interested in one or a few TFs within a network and for whom average performance across a large dataset is not particularly useful. The difference between the two is highlighted by our finding that two different performance parameters result in highly similar average performance, but radically different single TF performance profiles. As a result of these advantages the number of factors for which TG detection becomes highly performant (AUC values above 0.90) is increased from 5/34 to 19/34.

We attempted to address some of the questions facing regulatory bioinformatics. The major conclusions from our work are as follows. First, there is unlikely to be a single unifying CRM logic, at least at current levels of genome annotation resolution. We find that whereas some TFs perform optimally with single TFBS parameters (data not shown), others use clusters of homotypic TFBSs and still others use heterotypic TFBS clusters. Thus, a methodology that can determine the optimal approach per TF *a priori* is necessary for successful single TF based TG predictions. Second, the availability of multiple genomes is, in general, extremely useful for genome-wide TG prediction. One exception to this rule is that, contrary to conventional wisdom and several previous reports, additional genomes do not always result in better PWM building. The reason for this could be that PWMs that are built from sufficiently distinct binding sites (e.g., more than 20) already possess enough variation and do not benefit from the addition of more sites. A small amount of PWMs with very few, but highly conserved sites (e.g., HLHm5) do benefit from a phylogenetic extension.

The availability of multiple genomes becomes especially useful in two other ways. First, it improves the training of a CRM model from a set of known target regions, to discover sites that are both conserved and shared across this set. We have assessed whether such *de novo* discovered motifs could contribute to a better enhancer model for the TF. Second, it improves the scoring of a CRM model by taking advantage of functional enhancer conservation or TFBS conservation. We found no obvious explanation (e.g., in terms of correlations with TF families, with the conservation of known sites, or with the size of the TG set) why some TFs perform better with network-level conservation and others with motif conservation. When more *cis*-regulatory data becomes available as validation sets, for example through open community based annotation [45], a deeper investigation of this issue may become feasible.

Although several of the tested variables, most importantly the integration of multiple genomes, can result in significantly enhanced TG prediction accuracy, more work is needed to improve on this performance because only a portion of the true target enhancers could be detected. Again, it can be expected that performances will increase further, when more knowledge about regulatory regions emerges. For example, King et al. found recently that in vertebrates some regulatory regions correlate well with phastCons conservation scores (used for our motif conservation), while other regulatory regions correlate better with alignment-based scores that are corrected for background neutral substitution rates[46]. However, even with more advanced interspecies comparisons, on a genomic scale the true positive TF-TG interactions are spread out across many other high-scoring interactions. At present it is difficult to determine for a certain TF whether the other high scoring genes are also *bona fide* TGs, false positive predictions, or- most likely- a mix of both. Several ways can be envisioned to further improve the performance. For example, enhancer predictions can be combined with other data types to filter the ranked enhancers, such as GO terms, as we have shown for the TFs in Table 1, and/or large scale expression data, as we tested for the eye determination TF Ey. The benchmark dataset can be used in the future to evaluate novel methods for *de novo* motif discovery, enhancer prediction, or target gene prioritization.

In summary, we have tested several strategies and parameters for the computational prediction of TF-TG relations through TFBS and CRM detection. The selection of the best strategy for each individual TF, combined with the extensive use of multiple genomes during both the training and scoring of enhancer models results in a significant step forward in the bioinformatics to solving the architecture of gene regulatory networks.

## Materials and Methods

### Data

Our dataset consists of 166 TF-target relations for 34 transcription factors, generated by selecting all known TFs from FlyReg [20] that have minimally 3 distinct target genes (Table S1). Each TF-TG is represented by a test sequence, defined by selecting 1000 bp flanking sequence around only one of the TF-specific footprints around the TG. The ‘experimental’ PWMs are constructed by taking the best hit within each footprint after scoring with the corresponding matrices that were construced by Daniel Pollard using the MEME algorithm (http://rana.lbl.gov/dan/matrices.html). The scoring was done using Patser [47]. PWM scrambling is done by permuting the columns of a matrix, thereby conserving the A/T en C/G composition. 500 negative sequences are selected ad random from all 1 kb proximal sequences from the UCSC Table Browser [48]. Other negative sets that we tested are all the REDfly [31] enhancers with a maximal size of 1 kb (308 in total), extended in the genome to 1 kb, and 250 sequences of 1 kb surrounding a test sequence (125 on each side). Sets of orthologous sequences for positive and negative *Dmel* sequences are assembled using the liftOver utility of the UCSC Genome Browser [49]. Multiple output regions, due to homology to discontinuous regions, are all retained for training and scoring. Aligned sequences to *Dmel* TFBSs, used to build phyloPWMs, are also obtained through the UCSC liftOver utility. PhastCons conservation scores were downloaded from the UCSC download pages. Species and UCSC assemblies used throughout the analyses are *D. melanogaster* (dm2), *D. simulans* (DroSim1), *D. sechellia* (DroSec1), *D. yakuba* (DroYak1), *D. erecta* (DroEre2), *D. ananassae* (DroAna2), *D. pseudoobscura* (dp3), *D. persimilis* (DroPer1), *D. virilis* (DroVir2), *D. mojavensis* (DroMoj2), and *D. grimshawi* (DroGri1).

### Cross-validation

Methods for motif discovery, for CRM prediction, and for the use of multiple genomes are compared through leave-one-out cross-validation (LOOCV) (see Figure 1). For a regulon of *N* targets, an enhancer model is trained on *N*-1 positive regions of 1000 bp. The model is used to score the N^th^ (left-out) target region and a set of negative sequences. All sequences are then ordered by descending score and the rank of the left-out region is recorded. For one TF, this process (training, scoring, ranking) is repeated N times, each time leaving out another target region. The process is also repeated for each TF. This way, a total of 166 rank positions are obtained. By ordering these, and plotting them cumulatively, a kind of Receiver Operating Characteristic (ROC) is obtained. For different analysis methods, different curves are obtained that can be compared. Also, the area under this curve (AUC) is a measure for the overall detection performance integrating sensitivity and specificity values.

### Sequence scoring

The program Cluster-Buster [15] is used to score a sequence with a set of PWMs, using 1000 bp (the length of the test sequences) as range (-r option) for counting local nucleotide abundances. Order statistics are applied to integrate Cluster-Buster-based rankings on multiple genomes as described in [30] and in Figure 3B. STUBBMS is used with windowsize and shiftsize 1000, to give one score to the 1 kb test sequence. Gene Ontology statistics on lists of top scoring genes were calculated with the Generic GO Term Finder at http://go.princeton.edu/cgi-bin/GOTermFinder [50].

### Pattern discovery

For each LOOCV run, the Gibbs sampling program MotifSampler [38] was run 50 times for motifwidths 5, 6, 8, 10, 12, and 14, prior 0.2 and a 0^th^ order backgroundmodel from the whole 1 kb dataset. Resulting motifs were clustered and ranked according to information content as done in [40].

The program oligo-analysis [39] detects motifs in sequences by counting the occurrences of all oligonucleotides, and calculating their significance according to a background model, estimated by counting the occurrences of all oligonucleotides in all species-specific upstream sequences. Prior to their analysis, training sequences were purged with the program mkvtree [51] to discard internally repeated fragments. The genome-wide repetitive fragments were also masked for the pattern discovery step using UCSC Genome Browser RepeatMasker annotation (http://www.repeatmasker.org). Oligonucleotide occurrences of all sizes between 6 and 8 were counted on both strands, only considering the renewing occurrences (self-overlapping occurrences of a same word were discarded). The threshold of significance was set to 1, corresponding to an E-value of 1 false positive oligonucleotide per 10 training sets. Each run of oligo-analysis returns a set of over-represented oligonucleotides, which were used to construct a pseudo-PWM for each over-represented oligo with a value of 10 for the letters forming the word, and 0 for the other letters (see Note S1). To use oligo-analysis on multiple species, we detect over-represented words in each species separately, and use those to score the test regions of the respective species.

The PhyloGibbs program allows detecting motifs that are both conserved and shared across co-regulated sequences [41]. PhyloGibbs was run with parameters -D1 -m8 -z5 (5 motifs of width 8), -L“((DroGri1:0.7,(DroVir2:0.75,DroMoj2:0.75):0.7):0.5,((DroPer1:0.95,Dp3:0.95):0.7,(DroAna2:0.8,(DroEre1:0.82,(DroYak1:0.85,(Dm2:0.9,(DroSec1:0.95,DroSim1:0.95):0.9):0.85):0.82):0.8):0.7):0.5)” and whole chr2L as background sequence. Resulting matrices were compared with FlyReg matrices using the Kullback-Leiber distance [17].

## Supporting Information

Figure S1 Leave-one-out cross-validation performance for different negative sets. The rank of the positive “test” region (1 kb) within a set of negative sequences (all 1 kb) is plotted cumulatively. As negative sequences were used 500 randomly selected proximal promoter sequences, upstream of the annotated transcription start site (black curve) or 308 REDfly enhancers of maximally 1 kb length (blue curve), then all genomically extended to 1 kb, or 250 flanking sequences around the positive region (green curve), or 500 randomly generated sequences of 1 kb using a 5th order Markov model trained on all Dmel upstream sequences.
(0.15 MB PDF)

Figure S2 Cmparison of PhyloGibbs PWMs and real PWMs. All 166×5 motifs resulting from PhyloGibbs were compared to all 34 real PWMs, using the progam MotifComparison that implements the Kullback-Leiber distance between matrices [1]. Left column are real PWMs, middle column are matching PhyloGibbs motifs, and right column is the distance between both. Only eight real PWMs could be matched below distance threshold 1.0.
(0.70 MB PDF)

Figure S3 Overlap between Ey candidate targets obtained from microarray data and from genome-wide binding site prediction. At different cut-offs N (x-axis), the N top scoring Ey candidate targets, based on motif detection, are compared with the 188 Ey candidate targets obtained from gene expression studies [1]. The same is done for each of 100 randomized rankings (obtained by using a randomized Ey PWM). (A) For each cut-off value, the number of genes in common between the two sets is plotted on the y-axis. The values for the true Ey PWM are in blue, while the mean values of the randomized PWMs are in black. A 95% confidence interval is plotted in red dashed lines. (B) A p-value for each cut-off value is plotted on the y-axis. The p-values are calculated using a normal distribution based on the mean and standard deviation from the randomized rankings. The optimal p-value is obtained by using a cut-off value of 171.
(0.08 MB PDF)

Table S1 Dataset used in the study. 166 TF-target relations extracted from the FlyReg database [1]. For all factors with at least three different target genes, one footprint was chosen. 1000 bp flanking this footprint is used as training or test sequence in the cross-validation.
(0.11 MB PDF)

Note S1 Linking oligo-analysis output with Cluster-Buster input.
(0.07 MB PDF)
