# Supplementary material for: Fine-Tuning Enhancer Models to Predict Transcriptional Targets across Multiple Genomes
Source: PLoS One. 2007 Nov 7;2(11):e1115. doi: 10.1371/journal.pone.0001115 (PMC2047340; doi:10.1371/journal.pone.0001115)

Supplementary Figure 1: Leave-one-out cross-validation performance for different negative sets. The rank of the positive "test" region (1kb) within a set of negative sequences (all 1kb) is plotted cumulatively. As negative sequences were used 500 randomly selected proximal promoter sequences, upstream of the annotated transcription start site (black curve) or 308 REDfly enhancers of maximally 1kb length (blue curve), then all genomically extended to 1kb, or 250 flanking sequences around the positive region (green curve), or 500 randomly generated sequences of 1kb using a 5th order Markov model trained on all *Dmel* upstream sequences.

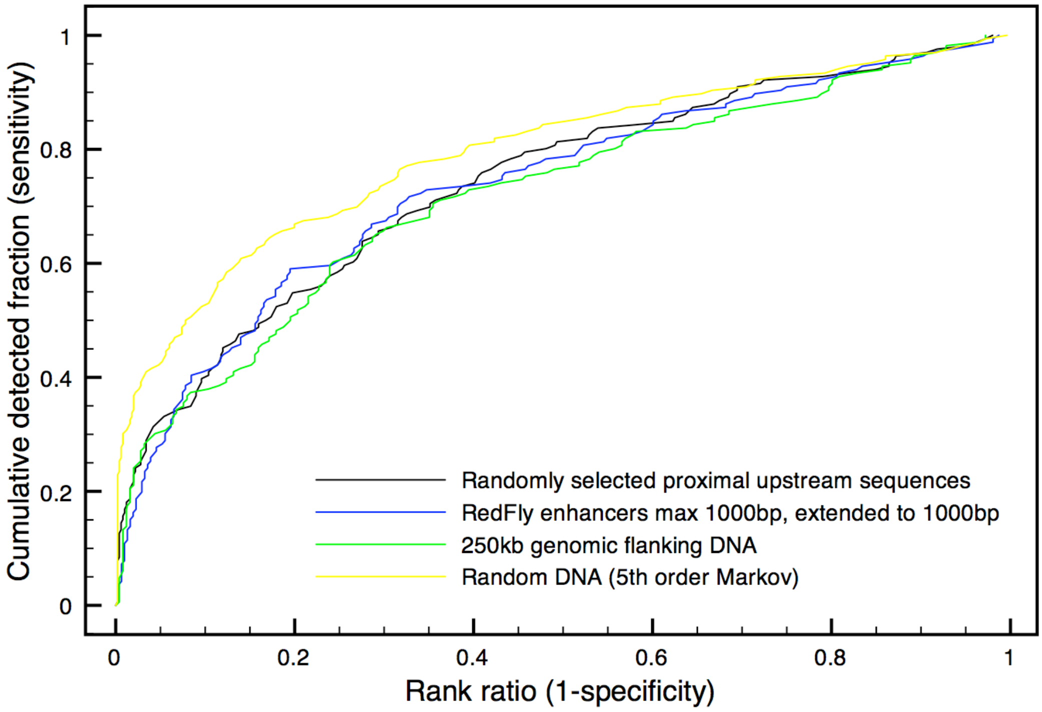

Supplement: Figure S1 — Leave-one-out cross-validation performance for different negative sets. The rank of the positive “test” region (1 kb) within a set of negative sequences (all 1 kb) is plotted cumulatively. As negative sequences were used 500 randomly selected proximal promoter sequences, upstream of the annotated transcription start site (black curve) or 308 REDfly enhancers of maximally 1 kb length (blue curve), then all genomically extended to 1 kb, or 250 flanking sequences around the positive region (green curve), or 500 randomly generated sequences of 1 kb using a 5th order Markov model trained on all Dmel upstream sequences. (0.15 MB PDF) [file pone.0001115.s001.pdf]
