# Supplementary material for: Fine-Tuning Enhancer Models to Predict Transcriptional Targets across Multiple Genomes
Source: PLoS One. 2007 Nov 7;2(11):e1115. doi: 10.1371/journal.pone.0001115 (PMC2047340; doi:10.1371/journal.pone.0001115)
Supplement: Figure S2 — Cmparison of PhyloGibbs PWMs and real PWMs. All 166×5 motifs resulting from PhyloGibbs were compared to all 34 real PWMs, using the progam MotifComparison that implements the Kullback-Leiber distance between matrices [1]. Left column are real PWMs, middle column are matching PhyloGibbs motifs, and right column is the distance between both. Only eight real PWMs could be matched below distance threshold 1.0. (0.70 MB PDF) [file pone.0001115.s002.pdf]

Supplementary Figure 2: comparison of PhyloGibbs PWMs and real PWMs. All 166x5 motifs resulting from PhyloGibbs were compared to all 34 real PWMs, using the progam MotifComparison that implements the Kullback-Leiber distance between matrices [1]. Left column are real PWMs, middle column are matching PhyloGibbs motifs, and right column is the distance between both. Only eight real PWMs could be matched below distance threshold 1.0.

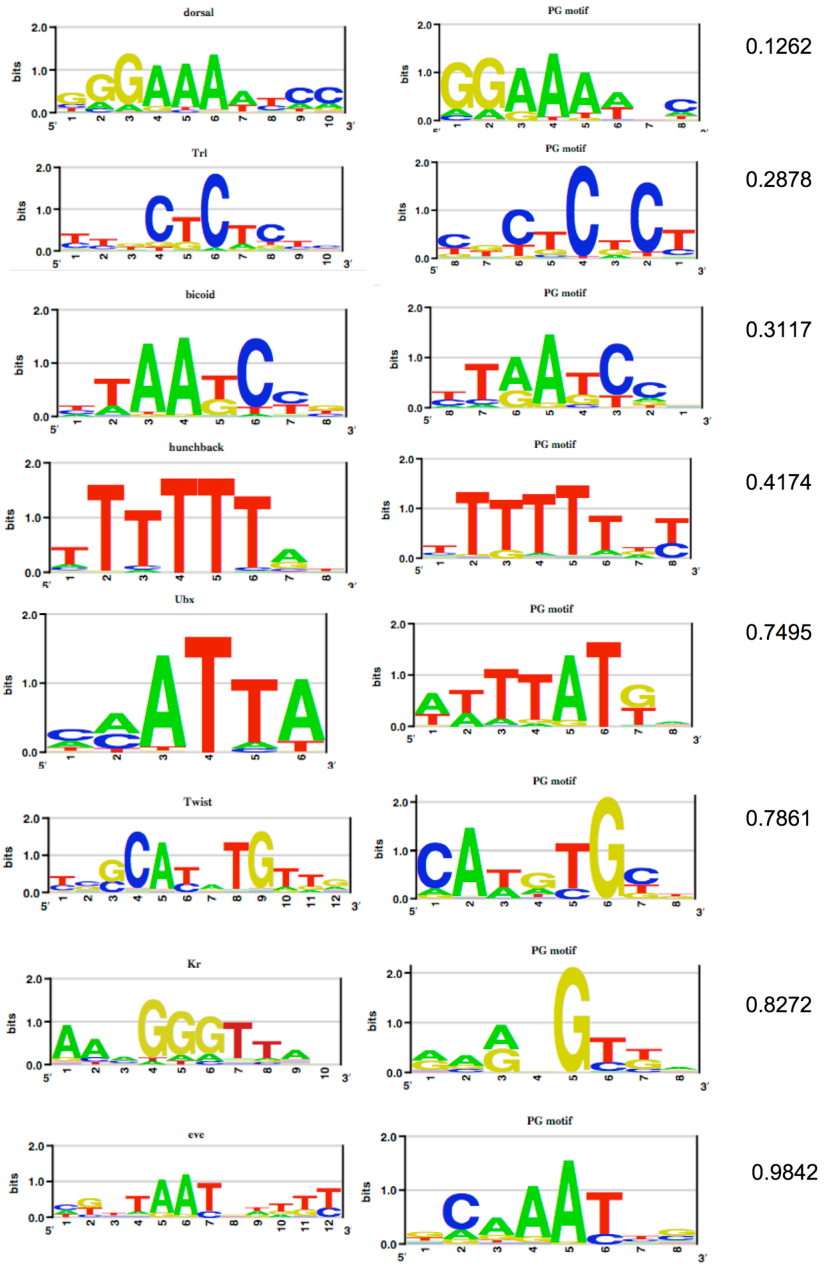

References

1. Aerts S, Thijs G, Coessens B, Staes M, Moreau Y, et al. (2003) Toucan: deciphering the cis-regulatory logic of coregulated genes. Nucleic Acids Res 31: 1753-1764.
