# Supplementary material for: Fine-Tuning Enhancer Models to Predict Transcriptional Targets across Multiple Genomes
Source: PLoS One. 2007 Nov 7;2(11):e1115. doi: 10.1371/journal.pone.0001115 (PMC2047340; doi:10.1371/journal.pone.0001115)
Supplement: Figure S3 — Overlap between Ey candidate targets obtained from microarray data and from genome-wide binding site prediction. At different cut-offs N (x-axis), the N top scoring Ey candidate targets, based on motif detection, are compared with the 188 Ey candidate targets obtained from gene expression studies [1]. The same is done for each of 100 randomized rankings (obtained by using a randomized Ey PWM). (A) For each cut-off value, the number of genes in common between the two sets is plotted on the y-axis. The values for the true Ey PWM are in blue, while the mean values of the randomized PWMs are in black. A 95% confidence interval is plotted in red dashed lines. (B) A p-value for each cut-off value is plotted on the y-axis. The p-values are calculated using a normal distribution based on the mean and standard deviation from the randomized rankings. The optimal p-value is obtained by using a cut-off value of 171. (0.08 MB PDF) [file pone.0001115.s003.pdf]

Supplementary Figure 3: Overlap between Ey candidate targets obtained from microarray data and from genome-wide binding site prediction

At different cut-offs *N* (x-axis), the *N* top scoring Ey candidate targets, based on motif detection, are compared with the 188 Ey candidate targets obtained from gene expression studies [1]. The same is done for each of 100 randomized rankings (obtained by using a randomized Ey PWM).

(A) For each cut-off value, the number of genes in common between the two sets is plotted on the y-axis. The values for the true Ey PWM are in blue, while the mean values of the randomized PWMs are in black. A 95% confidence interval is plotted in red dashed lines.

(B) A p-value for each cut-off value is plotted on the y-axis. The p-values are calculated using a normal distribution based on the mean and standard deviation from the randomized rankings. The optimal p-value is obtained by using a cut-off value of 171.

A

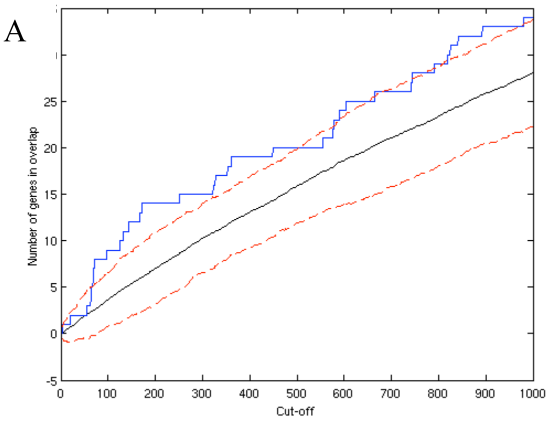

B

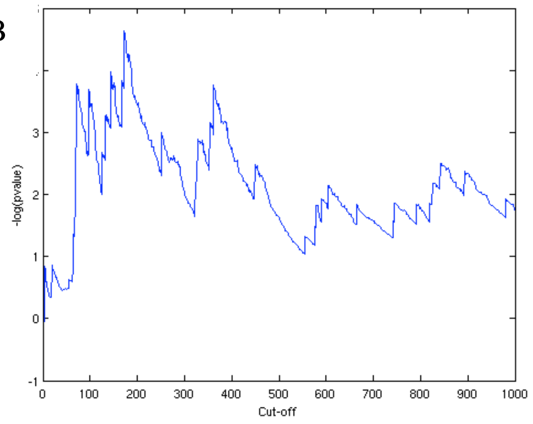

References

1. Ostrin JE, Li Y, Hoffman K, Liu J, Wang K, et al. (2006) Genome-wide identification of direct targets of the Drosophila retinal determination protein Eyeless. Genome Res 16: 466-476.
