# Supplementary material for: Fine-Tuning Enhancer Models to Predict Transcriptional Targets across Multiple Genomes
Source: PLoS One. 2007 Nov 7;2(11):e1115. doi: 10.1371/journal.pone.0001115 (PMC2047340; doi:10.1371/journal.pone.0001115)
Supplement: Table S1 — Dataset used in the study. 166 TF-target relations extracted from the FlyReg database [1]. For all factors with at least three different target genes, one footprint was chosen. 1000 bp flanking this footprint is used as training or test sequence in the cross-validation. (0.11 MB PDF) [file pone.0001115.s004.pdf]

Supplementary Table 1: Dataset used in the study.
166 TF-target relations extracted from the FlyReg database [1]. For all factors with at least three different target genes, one footprint was chosen. 1000bp flanking this footprint is used as training or test sequence in the cross-validation.

chromosomal location	factor->target	reference	FlyReg footprint ID
chr3L:6758912-6758933	vvl->vvl	PMID:8657157	FPID:000948
chr2R:8412592-8412614	vvl->vg	PMID:10862753	FPID:006301
chr3L:14043357-14043372	vvl->btl	PMID:9012536	FPID:002486
chr3R:2830985-2831027	Antp->Antp	PMID:2903553	FPID:004112
chr2L:21840625-21840641	Antp->tsh	PMID:7555708	FPID:002348
chr2R:1216997-1217034	Antp->ap	PMID:11262224	FPID:006193
chr3R:2758852-2758870	abd-A->Antp	PMID:8096172	FPID:006081
chr2L:21840625-21840641	abd-A->tsh	PMID:7555708	FPID:002347
chr2R:20311247-20311266	abd-A->Dll	PMID:1358457	FPID:006297
chr2L:2445883-2445893	abd-A->dpp	PMID:7906203	FPID:003637
chr3R:26676841-26676856	bcd->tll	PMID:8443106	FPID:006250
chr2R:5489661-5489670	bcd->eve	PMID:2026328	FPID:004117
chr3R:9720582-9720598	bcd->ems	PMID:11731230	FPID:003678
chr2R:20730450-20730474	bcd->Kr	PMID:2065664	FPID:003685
chr3L:20631194-20631232	bcd->kni	PMID:7617036	FPID:006051
chr2L:11455642-11455655	bcd->salm	PMID:9376314	FPID:006003
chr3L:8639161-8639170	bcd->h	PMID:9250684	FPID:005919
chr3R:4520376-4520389	bcd->hb	PMID:2911348	FPID:006413
chr3R:2758675-2758698	Ubx->Antp	PMID:8096172	FPID:006078
chr3R:12559839-12559931	Ubx->Ubx	PMID:2904838	FPID:006146
chr2L:21840625-21840641	Ubx->tsh	PMID:7555708	FPID:002346
chr2R:19816625-19816638	Ubx->betaTub60D	PMID:10628847	FPID:004395
chr2R:20311070-20311079	Ubx->Dll	PMID:1358457	FPID:006295
chr2L:11454562-11454576	Ubx->salm	PMID:12070087	FPID:006341
chr2L:2445883-2445893	Ubx->dpp	PMID:7906203	FPID:003638
chr3R:12559846-12559932	zen->Ubx	PMID:2569362	FPID:006148
chr3R:17497593-17497626	zen->DNApol-alpha180	PMID:7905482	FPID:006000
chr2L:13905028-13905048	zen->Ance	PMID:9053307	FPID:004210
chr2R:7044519-7044545	zen->en	PMID:2895896	FPID:006374
chr2R:15777850-15777895	zen->mus209	PMID:1968224	FPID:006352
chr2L:19115392-19115409	br-Z4->Ddc	PMID:8582299	FPID:005445
chrX:3105443-3105467	br-Z3->Sgs4	PMID:8062827	FPID:004472
chr3L:9354221-9354247	br-Z1->Hsp23	PMID:11583936	FPID:001199
chr3L:15622700-15622727	br-Z1->Eig71Eg	PMID:8954742	FPID:004527
chr3R:26677881-26677890	ttk->tll	PMID:12128207	FPID:005156
chr3R:2685246-2685265	ttk->ftz	PMID:1640455	FPID:004909
chr2R:5485830-5485843	ttk->eve	PMID:1372245	FPID:001791
chr3R:2579957-2579970	Mad->zen	PMID:11159914	FPID:006258
chr2R:8412626-8412634	Mad->vg	PMID:9230443	FPID:006302
chr3R:17209379-17209408	Mad->tin	PMID:9694800	FPID:000747
chr3R:12526877-12526902	tll->Ubx	PMID:8404855	FPID:002449
chr3R:9720620-9720633	tll->ems	PMID:11731230	FPID:003680
chr2R:20730327-20730343	tll->Kr	PMID:1348871	FPID:003682
chr3L:20630380-20630397	tll->kni	PMID:1546296	FPID:006030
chr3L:8639156-8639169	tll->h	PMID:9250684	FPID:005918
chr3R:4526653-4526687	tll->hb	PMID:7555732	FPID:005987

chr3R:2825018-2825059	Adf1->Antp	PMID:2318884	FPID:006446
chr2L:19116303-19116321	Adf1->Ddc	PMID:2318884	FPID:005464
chr2L:14615472-14615509	Adf1->Adh	PMID:2105454	FPID:005046
chr2L:2454657-2454685	Adf1->dpp	PMID:7791801	FPID:003665
chr3R:2689567-2689589	cad->ftz	PMID:2571934	FPID:004966
chr3L:20631234-20631246	cad->kni	PMID:7617036	FPID:006056
chr2L:11455649-11455657	cad->salm	PMID:9376314	FPID:006004
chr2L:3823210-3823236	pan->slp1	PMID:11076769	FPID:000600
chr2R:5497183-5497197	pan->eve	PMID:11783990	FPID:002461
chr3R:22997064-22997083	pan->Ser	PMID:14701680	FPID:006471
chr3R:2759092-2759112	ftz->Antp	PMID:1982071	FPID:006091
chr3R:12526852-12526902	ftz->Ubx	PMID:8404855	FPID:002447
chr2L:21840983-21840991	ftz->tsh	PMID:9431813	FPID:002358
chr3R:2684546-2684563	ftz->ftz	PMID:1976571	FPID:004895
chr2R:20564543-20564553	ftz->gsb	PMID:10885752	FPID:003455
chr2R:7041392-7041402	ftz->en	PMID:9043065	FPID:006389
chr3R:12636896-12636911	eve->abd-A	PMID:10644409	FPID:000264
chr3R:12559846-12559932	eve->Ubx	PMID:2569362	FPID:006147
chr2R:5485758-5485782	eve->eve	PMID:1671662	FPID:001787
chr2R:20564543-20564553	eve->gsb	PMID:10885752	FPID:003454
chr3R:17205861-17205897	eve->tin	PMID:9362473	FPID:002343
chr2R:7044519-7044545	eve->en	PMID:2895896	FPID:006375
chr2R:15777850-15777895	eve->mus209	PMID:1968224	FPID:006351
chr3L:1445543-1445553	twi->rho	PMID:1325394	FPID:000906
chr3R:12526729-12526748	twi->Ubx	PMID:8404855	FPID:002445
chr2L:15474246-15474260	twi->sna	PMID:1644293	FPID:006283
chr3R:8895841-8895847	twi->sim	PMID:9840810	FPID:004502
chr3R:17203832-17203870	twi->tin	PMID:9211899	FPID:000300
chr3R:12560183-12560204	Trl->Ubx	PMID:2897243	FPID:006155
chr3R:26677843-26677856	Trl->tll	PMID:8543159	FPID:005154
chr3R:7784091-7784111	Trl->Hsp70Ab	PMID:2781290	FPID:005504
chr3R:2912173-2912190	Trl->alphaTub84B	PMID:7984422	FPID:004152
chr2L:14615336-14615369	Trl->Adh	PMID:1408750	FPID:005041
chr3L:14722414-14722433	Trl->Trl	PMID:12200449	FPID:006166
chr2R:20734104-20734119	Trl->Kr	PMID:1985916	FPID:003002
chr3L:9351403-9351428	Trl->Hsp26	PMID:8474442	FPID:005512
chr3L:17584613-17584631	Trl->Eip74EF	PMID:2501151	FPID:005967
chr2L:2454724-2454732	Trl->dpp	PMID:7791801	FPID:003668
chr3R:12636468-12636491	Kr->abd-A	PMID:10644409	FPID:000260
chr3R:12598979-12598989	Kr->Ubx	PMID:1687458	FPID:005369
chr3L:21021992-21022005	Kr->ko	PMID:9311990	FPID:006319
chr2R:5489527-5489537	Kr->eve	PMID:2507923	FPID:004114
chr3L:20630372-20630390	Kr->kni	PMID:7617036	FPID:006028
chr2L:11455641-11455654	Kr->salm	PMID:9376314	FPID:006002
chr3L:8639314-8639324	Kr->h	PMID:9250684	FPID:005927
chr2R:7044647-7044657	Kr->en	PMID:1671661	FPID:006381
chr3R:4520680-4520692	Kr->hb	PMID:2797150	FPID:006422
chr3L:14063389-14063415	srp->Fbp1	PMID:10409761	FPID:005977
chrX:9898914-9898926	srp->Yp1	PMID:8524261	FPID:005548
chr2L:14616179-14616193	srp->Adh	PMID:8187633	FPID:005060
chrX:9898778-9898797	slbo->Yp1	PMID:7720712	FPID:005541
chr2R:19841709-19841731	slbo->slbo	PMID:1459454	FPID:006136
chr3L:14042329-14042342	slbo->btl	PMID:7671793	FPID:002483

chr3R:12598703-12598712	kni->Ubx	PMID:1687458	FPID:005367
chr2R:5487347-5487361	kni->eve	PMID:8626035	FPID:002266
chr2R:20730883-20730899	kni->Kr	PMID:1348871	FPID:003697
chr3L:8638499-8638509	kni->h	PMID:8186146	FPID:005955
chr3R:12560253-12560269	grh->Ubx	PMID:2606344	FPID:006161
chr3R:2581251-2581271	grh->zen	PMID:8543160	FPID:006279
chr2L:19116289-19116302	grh->Ddc	PMID:2792757	FPID:005463
chr3R:26677841-26677864	grh->tll	PMID:8543159	FPID:005153
chr3R:2688251-2688267	grh->ftz	PMID:2606344	FPID:005549
chr2L:2456664-2456671	grh->dpp	PMID:8543160	FPID:005241
chrX:10590262-10590283	Dref->ras	PMID:9819433	FPID:004539
chr3R:23064788-23064805	Dref->DNApol-alpha73	PMID:8662923	FPID:002339
chr3R:17497465-17497495	Dref->DNApol-alpha180	PMID:8093616	FPID:005998
chr3R:17458944-17458952	Dref->E2f	PMID:9748283	FPID:006068
chrX:226540-226558	HLHm5->ac	PMID:8078474	FPID:003024
chrX:265911-265932	HLHm5->l(1)sc	PMID:8078474	FPID:003027
chr3R:21865770-21865785	HLHm5->E(spl)	PMID:8078474	FPID:003017
chr3L:1445373-1445384	dl->rho	PMID:1325394	FPID:000901
chr3R:2581101-2581111	dl->zen	PMID:8344257	FPID:006272
chr2R:18552808-18552819	dl->twi	PMID:1648449	FPID:006449
chr2L:15475287-15475306	dl->sna	PMID:1644293	FPID:006285
chr2L:2456178-2456195	dl->dpp	PMID:8458580	FPID:005229
chr3R:19106285-19106304	ovo->orb	PMID:10637336	FPID:006366
chrX:8334700-8334725	ovo->otu	PMID:11290304	FPID:006363
chrX:6937187-6937207	ovo->Sxl	PMID:10637336	FPID:006349
chrX:4908875-4908894	ovo->ovo	PMID:9634487	FPID:001594
chr3R:12481457-12481463	z->Ubx	PMID:3145199	FPID:002906
chrX:2651308-2651314	z->w	PMID:3145199	FPID:006461
chr3L:17584613-17584629	z->Eip74EF	PMID:2501151	FPID:005968
chrX:2304146-2304152	z->z	PMID:3145199	FPID:002916
chr2L:2450873-2450879	z->dpp	PMID:3145199	FPID:002910
chr2R:5447128-5447137	tin->Mef2	PMID:9034334	FPID:005979
chr2R:5497332-5497339	tin->eve	PMID:11783990	FPID:002467
chr2R:19811988-19812004	tin->betaTub60D	PMID:10588882	FPID:000670
chr3R:17209344-17209352	tin->tin	PMID:9694800	FPID:000745
chr3R:12636938-12636961	gt->abd-A	PMID:10644409	FPID:000267
chr2R:5489681-5489707	gt->eve	PMID:2026328	FPID:004119
chr2R:20730905-20730921	gt->Kr	PMID:1576969	FPID:003700
chr3L:20630775-20630787	gt->kni	PMID:7617036	FPID:006044
chrX:226540-226558	E(spl)->ac	PMID:8078474	FPID:003023
chrX:249926-249967	E(spl)->sc	PMID:9649507	FPID:004534
chrX:265911-265932	E(spl)->l(1)sc	PMID:8078474	FPID:003026
chr3R:21865770-21865785	E(spl)->E(spl)	PMID:8078474	FPID:003016
chrX:7375592-7375606	sd->ct	PMID:11303087	FPID:001396
chr2R:19849908-19849934	sd->bs	PMID:9869643	FPID:000418
chr2R:8412378-8412389	sd->vg	PMID:11303087	FPID:006298
chr3L:20640791-20640809	sd->kni	PMID:12466192	FPID:001390
chr2L:11454575-11454582	sd->salm	PMID:11303087	FPID:006342
chr3R:12526803-12526815	en->Ubx	PMID:8404855	FPID:002446
chrX:1996382-1996439	en->ph-p	PMID:7600986	FPID:006306
chr3R:2692104-2692143	en->ftz	PMID:3046753	FPID:005108
chr4:80104-80117	en->ci	PMID:7600980	FPID:006198
chrX:1980286-1980378	en->ph-d	PMID:7600986	FPID:006317

chr2R:7041388-7041401	en->en	PMID:2573829	FPID:006388
chr2L:2471211-2471220	en->dpp	PMID:7713429	FPID:006408
chr3R:2580050-2580066	brk->zen	PMID:11159914	FPID:006261
chr3R:2507228-2507244	brk->lab	PMID:11080162	FPID:005392
chrX:4231705-4231722	brk->bi	PMID:11080162	FPID:000446
chr3R:12636602-12636615	hb->abd-A	PMID:10644409	FPID:000261
chr3R:12526647-12526665	hb->Ubx	PMID:8404855	FPID:002444
chr2R:5487362-5487372	hb->eve	PMID:2507923	FPID:002267
chr2R:20730350-20730404	hb->Kr	PMID:2065664	FPID:003683
chr3L:20630376-20630390	hb->kni	PMID:7617036	FPID:006029
chr2L:11456121-11456136	hb->salm	PMID:9376314	FPID:006017
chr3L:8638364-8638375	hb->h	PMID:8186146	FPID:005952
chr2R:7044664-7044674	hb->en	PMID:1671661	FPID:006382
chr3R:4520496-4520512	hb->hb	PMID:2797150	FPID:006416

References

1. Bergman MC, Carlson WJ, Celniker ES (2005) Drosophila DNase I footprint database: a systematic genome annotation of transcription factor binding sites in the fruitfly, Drosophila. Bioinformatics 21: 1747-1749.
