# Supplementary material for: Fine-Tuning Enhancer Models to Predict Transcriptional Targets across Multiple Genomes
Source: PLoS One. 2007 Nov 7;2(11):e1115. doi: 10.1371/journal.pone.0001115 (PMC2047340; doi:10.1371/journal.pone.0001115)
Supplement: Note S1 — Linking oligo-analysis output with Cluster-Buster input. (0.07 MB PDF) [file pone.0001115.s005.pdf]

Supplementary Note 1: Linking oligo-analysis output with Cluster-Buster input

Each run of oligo-analysis [1] returns a set of over-represented oligonucleotides, not position weight matrices (PWM), while the program Cluster-Buster requires PWMs as input. To solve this, we investigated three different ways to score a test sequence. First, a test sequence was scanned with the program dna-pattern to all occurrences of oligonucleotides found to be significant in the training sequences. Each occurrence was weighted according to the significance score obtained in the training set, and the weights of occurrences were summed to give a global weight to the test sequence. Second, for each regulon the significant oligonucleotides were assembled with the program pattern-assembly, with at most 1 mismatch and 1 flanking base. A set of oligonucleotides can result in one or several assemblies. Each assembly is converted to a position-specific scoring matrix, by selecting, for each position of the matrix, the nucleotides found in significant oligonucleotides at the corresponding position of the assembly. The score of each cell of the matrix corresponds to the significance of the most significant oligonucleotide having that residue at that position of the assembly. The resulting matrix is called a significance matrix. This significance matrix was used as a pseudo weight matrix to scan the training sequences in order to extract all the instances of the motif. These instances were then aligned and converted to a new matrix, where each cell represents the frequency of the corresponding residue at the corresponding position of the collected sites. This second matrix is called a count matrix. Each result of oligo-analysis was thus converted to one or several count matrices which were given as input to Cluster-Buster to assign a score to the test sequence. Third, a pseudo-PWM was constructed for each over-represented oligo with a value of 10 for the letters forming the word, and 0 for the other letters. The third option yielded the best results and was used throughout the analyses.

References

1. van Helden J, Andre B, Collado-Vides J (1998) Extracting regulatory sites from the upstream region of yeast genes by computational analysis of oligonucleotide frequencies. J Mol Biol 281: 827-842.
